# Low-grade myofibroblastic sarcoma of the orbit: A case report and literature review

**DOI:** 10.1097/MD.0000000000009172

**Published:** 2017-12-22

**Authors:** Shikun Zhang, Ying Ma, Tie Ma, Zhiming Wang

**Affiliations:** a Department of Oral and Maxillofacial Surgery; b Department of Pathology, Shengjing Hospital of China Medical University, Shenyang, China.

**Keywords:** clinical characteristics, low-grade myofibroblastic sarcoma, mesenchymal tumor, orbit

## Abstract

**Rationale:**

Low-grade myofibroblastic sarcoma (LGMS) is a malignant lesion composed of myofibroblasts. It is an uncommon tumor of unknown etiology that mainly develops in the bone or soft tissue and is most often reported in the head and neck, particularly in the tongue and oral cavity.

**Patient concerns:**

A 2-year-old girl, previously well and with no significant medical history or family history of other diseases, presented with a 2-week painless swelling of the right orbit.

**Diagnoses:**

Preoperative computed tomography (CT) revealed a large homogeneous enhanced mass, 21 × 13 mm in size, located on lateral wall of the right orbit with bone absorption. The mass was resected and histopathological examination revealed LGMS of the orbit.

**Interventions:**

On May 2016, she underwent surgery without the additional postoperative treatment.

**Outcomes:**

The patient's postoperative course was uneventful, and was discharged on the 6th day after surgery. During a year follow-up period, there was no recurrence of the postoperative CT. The patient and her family were satisfied with the result of the surgery.

**Lessons:**

Based on clinical characteristics and postoperative CT, we considered the mass may be a benign tumor. We completely resected along the capsule without an extensive surgical margin. However, postoperative histopathology diagnose LGMS, which shows a strong potential for local recurrence and vascular invasion. So we should close observation of the patient's symptoms and sign. If the tumor has invaded adjacent tissues, we will use adjuvant chemotherapy or radiotherapy.

## 1 Introduction

Myofibroblastic sarcoma was reported as a distinct entity in 1998 by Mentzel.^[1]^ Low-grade myofibroblastic sarcoma (LGMS) is an uncommon solid tumor of mesenchymal origin that usually develops in the head and neck.^[2]^ LGMS consists mainly of myofibroblasts, which are spindle-shaped mesenchymal cells with ultrastructural features of both fibroblasts and smooth muscle cells.^[3]^ Some myofibroblastic tumors have relatively benign or only low-grade malignant characteristics.^[4]^ LGMS has been most frequently reported in adult men,^[5]^ and has been found in a wide variety of tissues, including skin, breast, vulva, salivary glands, parapharyngeal space, jaw, larynx, nasal cavity/paranasal sinuses, soft tissue of the cheek, piriform fossa, soft palate, and tongue.^[6–18]^ Reports of LGMS of the orbit are extremely rare. Here, the authors report a case of a 2-year-old girl with LGMS of the orbit and discuss its clinical manifestations, pathological characteristics, diagnosis, therapy, and prognosis. The previous literature is reviewed.

## 2 Case report

**Figure 1:**
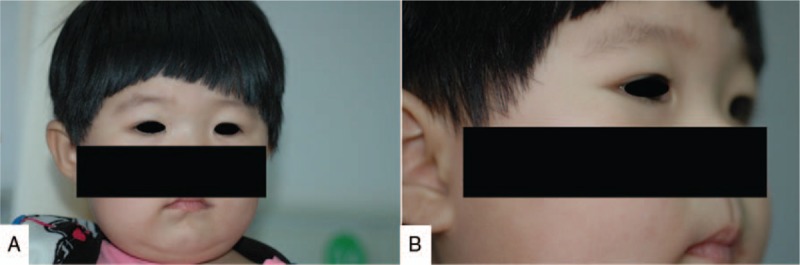
Preoperative photographs. The mass is located at the lateral aspect of the right orbit. A, Front view. B, Lateral view.

**Figure 2:**
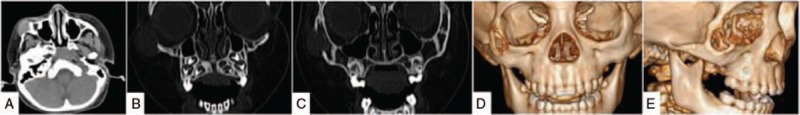
Three-dimensional computed tomography showing the soft tissue mass on the lateral wall of the right orbit. The density is uniform and the size is approximately 21 × 13 mm. There is bony destruction extending to the lateral wall of the right orbit from the zygomatic bone. A, Axial view. B and C, Coronal view. D and E, Three-dimensional reconstruction.

This study was approved by the Ethics Committee of Shengjing Hospital of China Medical University. Written informed consent was obtained from the patient's parent. A 2-year-old girl, previously well and with no significant medical history or family history of other diseases, presented with a 2-week painless swelling of the right orbit (Fig. 1A and B). On physical examination a palpable mass about the size of a quail egg was found to be located at the right orbit. The mass had unclear boundaries, was fairly immobile, and was not tender. Bilateral vision and eye movements were unaffected. Computed tomography (CT) showed a large homogeneous enhanced mass, 21 × 13 mm in size, located on lateral wall of the right orbit with bone absorption (Fig. 2A–E).

**Figure 3:**
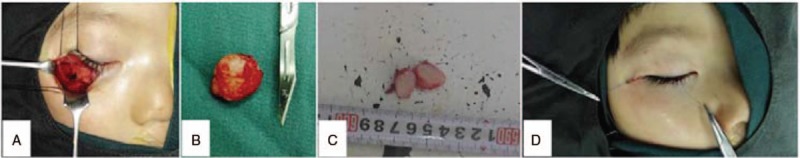
Intraoperative findings: (A) incision was made via the lower eyelid. The mass was located on the lateral right orbit. There was visible destruction of the bony wall of the right orbit. B, The mass was completely encapsulated and measured 2.0 × 1.5 × 1.5 cm. C, The tumor was uniform on transection and exhibited a yellowish and gray surface with a firm and rubbery texture. D, Suturing the incision.

The patient underwent open surgery under general anesthesia for excision of the mass, which was exposed via a right lower eyelid approach. The mass had an intact capsule measuring 2.5 × 2.5 × 1.5 cm and was completely resected along the capsule. Macroscopically, the tumor was round, firm, smooth, and well circumscribed, measured approximately 2.0 × 1.5 × 1.5 cm, and had a yellowish-gray and homogeneous surface on transection (Fig. 3A–D).

**Figure 4:**
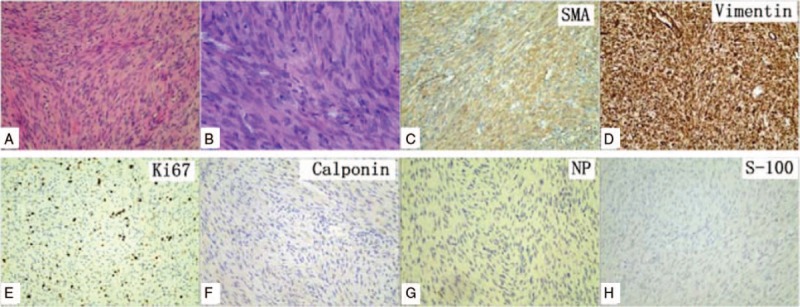
A and B, At lower magnifications, the tumor was composed of loosely arranged cells in a myxoid background (hematoxylin and eosin). A, Magnification × 100. B, Magnification ×400. C–H, On immunostaining, cells were strongly positive for smooth muscle actin (SMA) and vimentin, the Ki-67 index was >5%, and calponin, NP, and S-100 were negative.

Histopathologic examination confirmed the diagnosis of LGMS. Microscopic examination showed spindle-shaped tumor cells that were arranged in a fascicular or storiform pattern infiltrating the adjacent bone trabecula. The cells were demonstrated nuclear atypia with mitotic figures. The cytoplasm was acidic and the stroma consisted of a small number of collagen fibers. On immunohistochemical staining, the spindle-shaped cells were strongly and diffusely positive for smooth muscle actin (SMA) and vimentin (Fig. 4C and D). The Ki-67 index was >5% (Fig. 4E), and staining for calponin, NP, and S-100 were negative (Fig. 4F–H).

**Figure 5:**
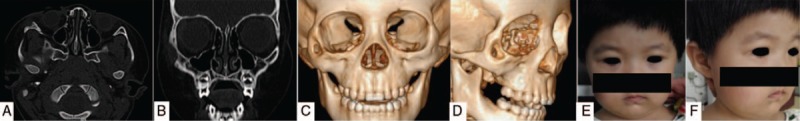
Follow-up evaluation at 3 months postoperatively. There is no evidence of recurrence and the affected area of bone is healing. A, Axial view. B, Coronal view. C and D, Three-dimensional reconstruction. E and F, Frontal and right lateral photographs of the patient.

**Figure 6:**
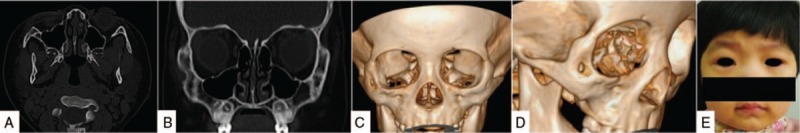
The patient remains recurrence-free at a year postoperatively, with additional healing of the affected bone. A, Axial view. B, Coronal view. C and D, Three-dimensional reconstructions. E, Frontal-view photograph of the patient.

The postoperative course was uneventful and the patient did not receive adjunctive therapy. The patient was discharged on the 14th day after surgery. No additional treatment was necessary, and there was no recurrence or metastasis of the postoperative CT during a year follow-up period (Figs. 5 and 6).

## 3 Discussion

The World Health Organization classification of soft-tissue tumors describes LGMS as an atypical myofibroblastic proliferation with fibromatosis-like features that most commonly develop in the head and neck.^[19]^ LGMS is a low-grade mesenchymal malignancy composed of myofibroblasts that can occur in submucosal and subcutaneous tissues, deep soft tissues, and intraosseous areas.^[20]^ Myofibroblasts are modified fibroblasts that are morphologically and functionally similar to both fibroblasts and smooth muscle cells.^[21]^ They were first reported in granulation tissue and can also arise in the stroma of normal, inflammatory, neoplastic, or reactive soft tissue lesions.^[3,22]^ LGMS shows a strong potential for local recurrence, while vascular invasion and malignant transformation occur only occasionally.^[23]^ In some cases, LGMS has an incomplete capsule and may infiltrate adjacent fibrous tissue, fat, or skeletal muscle.^[21]^

While LGMS is most commonly found in the head and neck, particularly in the tongue and oral cavity, it has also been reported in almost every other organ.^[24]^ The mean age of affected patients is 40 years, ranging from 4 to 85 years, and a preponderance of adult men is reported.^[25]^ The tumors generally present as a painless swelling or a slow-growing mass with a relatively indolent course.^[26]^ Some patients may present with pyrexia, chills, leukocytosis, or meningeal irritation.^[23]^

Although preoperative CT or magnetic resonance imaging is essential for diagnosis, LGMS is hardly diagnosed definitely by them. In vivo molecular imaging is an innovative and cornerstone method for clinical diagnosis for tumors. The new method collects information at the molecular and cellular levels in humans.^[27]^ Therefore, new molecular imaging may be an effective method for preoperative diagnosis, therapy as well as prognosis in malignant tumors, especially occurred in the head and neck regions. Of molecular imaging methodologies, 3 main techniques are intriguing. The first one is nuclear medical imaging, in which radioactive molecular probe is utilized and its distribution and kinetics are measured by positron emission tomography (PET) and single-photon emission computed tomography.^[27]^ Probes for nuclear molecular imaging were developed to image intact function difference between wild-type and the transduced type F98 gliomas in cat brain. ^18^F-labeled amino acids for PET/CT imaging were utilized to show elevated amino acid metabolism in gliomas, neuroendocrine tumors, prostate cancer, and breast cancer.^[27–29]^ A number of amino acid PET radiotracers were helpful in pretreatment evaluation of pediatric low-grade gliomas.^[30]^ The second one is optical molecular imaging, in which a fluorescent probe is used to provide real-time imaging of distribution and kinetics. Although high spatial resolution could be obtained inexpensively, the imaging region is limited to the body surface.^[31]^ The third one is photoacoustic imaging, which could detect highly sensitive and high-resolution photoacoustic signals in deep tissues.^[32]^ Thus, we speculated that new molecular imaging techniques could be helpful for preoperative diagnosis, therapy, and prognosis estimate in LGMS. Unfortunately, due to the lack of the new molecular imaging system, this patient was not performed these specific examinations before surgery. Because cytogenetic and molecular genetic alterations of LGMS are presently obscure, in vivo molecular imaging could be tested in this disease in the future.

A definitive diagnosis of LGMS is made mainly on the basis of histopathological findings and immunohistochemical analyses.^[33]^ Histologically, LGMS is mainly composed of slender spindle cells showing low mitotic activity and variable nuclear pleomorphism.^[24]^ The nucleus has been described as fusiform, slender, and undulate.^[6]^ The tumor cells are arranged in sheet-like interlacing fascicles or storiform patterns, and have eosinophilic cytoplasm with sparse inflammatory lymphocytic and plasmacytic infiltration.^[4]^ The interstitial tissue may consist of hyalinized collagen fibers.^[34]^ Immunohistochemical staining may be positive, to some degree, for SMA, muscle specific antigen, fibronectin, desmin, calponin, and vimentin. CD34 and CD99 are also positive in a small fraction, while anaplastic lymphoma kinase (ALK), S-100, and epithelial markers, including cytokeratin and epithelial membrane antigen, and laminin are generally negative.^[4,6,35]^

Surgery is the primary treatment modality for LGMS.^[36]^ Because LGMS may have a tendency for invasive growth and local recurrence, an extensive surgical margin should be recommended,^[37]^ although in our case, the mass was completely encapsulated. Complete resection of the primary lesion and local recurrent lesions is essential, but the optimal extent of resection has yet to be determined. Some authors have recommended adjuvant chemotherapy and radiotherapy, particularly if the tumor has invaded adjacent tissues or the lymphatic or hematological metastasis is evidenced.^[38]^ However, other reports have indicated that myofibrosarcoma is poorly responsive to radiation therapy, and that the cure rate with chemotherapy is uncertain.^[39]^ Thus, the best method of treatment requires further prospective investigation. We did not recommend postoperative chemo- or radiotherapy for the present patient because of concerns regarding side effects and preservation of her eyesight.

The differential diagnosis of LGMS includes a variety of malignant or benign lesions, including fibrosarcoma, leiomyosarcoma, inflammatory myofibroblastic tumor (IMT), nodular fasciitis, and fibromatosis.^[40]^ Fibrosarcomas are composed of malignant spindle cells showing fibroblastic differentiation, which are distinguished from myofibroblasts by the absence of immunohistochemical evidence of fibronectin, SMA, and calponin.^[41]^ Leiomyosarcoma is characterized by alternate fascicles of cells with marked cytological pleomorphism that show immunoreactivity for desmin, h-caldesmon, and occasionally keratin, without evidence of immunoreactivity for fibronectin.^[42]^ Nodular fasciitis is less cellular and uniform than LGMS, and has a heterogeneous appearance with cellular, myxoid, and fibrous areas.^[37]^ Fibromatosis may display a prominent nodularity, and tends to infiltrate adjacent tissues.^[37]^ The tumor cells are lacking of atypia and mitotic figures, and are positive only for vimentin.^[20]^ Finally, LGMS is most easily confused with IMT. IMT is mainly composed of myofibroblastic spindle cells mixed with prominently lympho- and plasmacytic infiltration, and is positive for ALK or cytokeratin.^[43]^ while LGMS has a more uniform histological pattern, with a greater degree of nuclear atypia and more mitotic figures, and it is negative for ALK and cytokeratin at immunohistochemistry.^[8]^

## 4 Conclusion

In the present report, we describe a rare case of LGMS in the wall of the right orbit in a 2-year-old girl. The tumor was completely encapsulated and followed a relatively indolent course. Complete resection was performed. The prognosis was good, and at a year after surgery the patient remained recurrence-free. LGMS presents many diagnostic challenges, particularly in the differentiation of a low-grade malignant process from an intermediate-grade malignant transformation. In vivo molecular imaging may be an effective method for diagnosis and postoperational follow-up for LGMS, and should be further evaluated.
